# Metabolites and proteins may mediate the relationship between diet quality and insulin sensitivity in young adult cohort

**DOI:** 10.1007/s40200-026-01918-3

**Published:** 2026-02-26

**Authors:** Elizabeth Costello, Jesse A. Goodrich, Brittney O. Baumert, Shiwen Li, Shudi Pan, William B. Patterson, Douglas I. Walker, Sarah Rock, Frank D. Gilliland, Michael I. Goran, Max T. Aung, Sandrah P. Eckel, Tanya L. Alderete, Zhanghua Chen, David V. Conti, Lida Chatzi

**Affiliations:** 1 Department of Population and Public Health Sciences, University of Southern California, Los Angeles, CA USA; 2 Department of Public Health Sciences, Thompson School of Social Work and Public Health, University of Hawaii at Manoa, Honolulu, HI USA; 3 Department of Integrative Physiology, University of Colorado Boulder, Boulder, CO USA; 4 Department of Biomedical Informatics, University of Colorado School of Medicine, Aurora, CO USA; 5 Gangarosa Department of Environmental Health, Emory University, Atlanta, GA USA; 6 Department of Pediatrics, Children’s Hospital Los Angeles, The Saban Research Institute, Los Angeles, CA USA; 7 Department of Environmental Health and Engineering, Johns Hopkins Bloomberg School of Public Health, Baltimore, MD USA; 8 Department of Biostatistics and Informatics, Colorado School of Public Health, Aurora, CO USA

**Keywords:** Insulin sensitivity, Diet, Metabolomics, Proteomics, Multi-omics study

## Abstract

**Purpose:**

Poor diet quality is a known risk factor for type 2 diabetes and related outcomes, including declines in insulin sensitivity. Biological changes that occur in response to diet may explain this relationship.

**Methods:**

This study was conducted in a cohort of young adults (the MetaAIR study, *n* = 77, 52% female, 57% Hispanic). High dimensional mediation analyses (HIMA) were performed to identity potential metabolite, protein, and miRNA mediators of the relationship between the Healthy Eating Index-2015 (HEI) and insulin sensitivity (Matsuda Index) over a four year follow up period. Features identified by HIMA and significant after correction for multiple comparisons (q < 0.05) were assessed using causal mediation analyses. The indirect effects and proportion mediated by each feature were calculated independently.

**Results:**

Each point increase in HEI was associated with a 0.051 (95% CI:0.004, 0.098) point increase in Matsuda Index. Four potential mediators were identified using HIMA: three metabolites (5Z,8Z,11Z-eicosatrienoic acid, pipecolic acid, and biotin), and one protein (F9). Each of these features exhibited a positive indirect effect and independently mediated between 35 and 43% of the total effect. No miRNAs were selected as potential mediators.

**Conclusion:**

These findings suggest that specific metabolites and proteins may mediate the association between diet and diabetes-related outcomes such as declines in insulin sensitivity, likely through pathways related to inflammation. F9, biotin, pipecolic acid, and 5Z,8Z,11Z-eicosatrienoic acid may be potential targets for monitoring efforts for diet adherence or disease prevention.

**Supplementary Information:**

The online version contains supplementary material available at 10.1007/s40200-026-01918-3.

## Introduction

Young-onset type 2 diabetes is a health condition of increasing concern, as the number of adolescents and young adults at risk for this condition grows [1]. Globally, the incidence rate of type 2 diabetes in youth has increased from 117 to 183 per 100,000 people between 1990 and 2019 [2]. In the United States, the incidence rate in adolescents has increased by over 5% each year between 2002 and 2018 [3], while type 2 diabetes-related mortality has been increasing at a greater rate in adults between aged 25–34 compared to older adults [4]. Because health outcomes for youth with type 2 diabetes are more severe than those diagnosed in later adulthood [5], there is a need for greater understanding of the complex mechanisms underlying type 2 diabetes disease progression and development. Declines in insulin sensitivity reflects a metabolic disruption leading to glucose intolerance and greatly increases the risk for type 2 diabetes, and often can be detected well in advance of a type 2 diabetes diagnosis [6]. Understanding and targeting this condition before progression to type 2 diabetes may prevent later disease and disability.

In youth, hyperinsulinemia appears to be more rapid than in older adults, and the progression to diabetes follows more quickly [7], highlighting the need for early and effective intervention. Poor diet is a significant risk factor for type 2 diabetes and its precursor conditions, and improving diet quality is an initial goal for clinicians and dieticians treating patients at risk. Diets high in fruits, vegetables, and fiber, and low in added sugars, saturated fat, and refined grains are recommended by the United States Department of Agriculture (USDA) Dietary Guidelines [8], and reduce the risk for type 2 diabetes in all populations [9, 10]. Advances in omics technologies have made it possible to evaluate the direct impact of dietary intake on biological processes, including those that might lead to the development of type 2 diabetes and other metabolic diseases [11]. It is hypothesized that some or all of the effects of dietary intake on metabolic disease may be mediated by omics features, such as those from the metabolome, proteome, and transcriptome [12–14]. Each omics layer influences others, both upstream and downstream; micro RNA (miRNA) and messenger RNA (mRNA) translation promote or suppress protein synthesis [15], while proteins regulate biological processes that produce metabolites [16]. Proteins decompose into metabolites, and metabolic products are involved in post-translational protein activity either as products (of enzymatic reactions, for instance) or as cofactors or signaling molecules [16]. Due to the biological interactions occurring between all omics layers, multi-omics integration analyses offer an opportunity to investigate relationships between omics features and identify underlying mechanisms of disease development.

A mediation analytical approach may provide insight into the molecular mechanisms underlying disease development and the biological effects of an exposure on disease risk. In a causal mediation framework, the total effect of an exposure on an outcome is decomposed into the “indirect” effect of a single intermediate on the causal pathway, and the remaining “direct” effect of the exposure [17]. High dimensional mediation analysis, with potentially thousands of omics features to act as mediators, may determine the extent to which the effects of a given exposure are explained by a collection of biological features along the causal path between an exposure (lifestyle factors, treatments, or other interventions) and health outcomes (insulin resistance, prediabetes, or type 2 diabetes) [18]. Some previous work has identified omics features as potential mediators between lifestyle factors and type 2 diabetes [19, 20], though to date these analyses have utilized only one omics layer, have included only middle aged or older adults, and have been conducted in populations with limited ethnic diversity. The present study aims to address these gaps by investigating the potential for multi-omics mediation in a unique cohort of primarily Hispanic young adults.

We aim to determine the extent to which the relationship between diet and markers of insulin sensitivity are mediated by features from three closely connected omics layers. We have previously shown that high-quality diet is protective against prediabetes and is associated with glucose homeostasis in this cohort [9]. Identifying potential mediating biomarkers in this population, composed of youth at risk for type 2 diabetes due to a history of overweight or obesity and Hispanic ethnicity, may provide insight into intervention strategies, novel methods of risk prediction, or mechanisms of type 2 diabetes development. Thus, we assess the longitudinal relationship between diet and markers of insulin sensitivity, measured via two-hour oral glucose tolerance test, as well as the mediating effects of features within three omics layers: miRNAs, proteomics, and metabolomics.

## Research design and methods

### Study population

Between 2014 and 2018, 155 participants (age 17–22) were recruited from the Children’s Health Study (CHS) in Southern California for the MetaAIR study [21], an observational study of risk factors for diabetes and other metabolic conditions in early adulthood. Participants were invited to the study if they had a history of overweight or obesity in early adolescence. Exclusion criteria were: diagnosis of type 1 or type 2 diabetes, diagnosis of another serious medical condition (i.e., cancer), and use of medications known to influence glucose metabolism. Between January 2020 and March 2022, 140 MetaAIR participants were invited to participate in a follow up visit (the “MetaCHEM” study), and 85 participants completed the second visit [9]. Both study visits took place at the Diabetes and Obesity Research Institute at the University of Southern California (USC). This study was approved by the USC Institutional Review Board and written informed consent was obtained from participants (and their guardians, if under age 18) at both visits.

### Diet assessment

At the first visit, participants completed two 24-hour dietary recalls on two nonconsecutive days, one weekday and one weekend day. Recalls were completed by trained interviewers using the Nutritional Data System for Research (NDSR) software version 14 (University of Minnesota, Minneapolis, MN). Recalls were administered in person for the first recall and over the phone for the second recall. These recalls were used to calculate the Healthy Eating Index 2015 (HEI), which measures adherence to the USDA 2015 Dietary Guidelines. This index consists of thirteen components that, when summed, produce a final score between 0 and 100. The components of the HEI are: total fruit (range: 0–5), whole fruit (0–5), total vegetables (0–5), greens and beans (0–5), whole grains (0–10), dairy (0–10), total protein foods (0–5), seafood and plant proteins (0–5), fatty acids (0–10), refined grains (0–10), sodium (0–10), added sugar (0–10), and saturated fats (0–10) [8]. The fruit, vegetable, greens and beans, grains, dairy, protein, and sodium components scores are determined based on the amount consumed per 1000 kilocalories. HEI was calculated separately for each recall, and the average of the two recalls was used in the analysis unless a participant did not complete the second recall.

### Outcome assessment

A two-hour oral glucose tolerance test (OGTT) was performed at the baseline and follow-up visits, using a glucose load of 75 g of glucose per kg of body mass (max 75 g). Blood samples were taken before the OGTT and at 30-, 60-, 90-, and 120 min post-glucose challenge. Glucose and insulin concentrations at each time point were measured in plasma. The OGTT was not completed if participants had a fasting glucose value greater than 126 mg/dL, measured by glucometer, due to safety concerns.

The homeostatic model assessments for beta cell function and insulin resistance (HOMA-β and HOMA-IR) were calculated to assess beta-cell function and insulin resistance, respectively, using the following formulas [22]:

$$\:\mathrm{HOMA-IR}=\:\frac{fasting\:insulin\:x\:fasting\:glucose}{22.5}$$

$$\:\mathrm{HOMA-}\beta\:=\frac{20\:x\:fasting\:insulin}{fasting\:glucose-3.5}$$

Where units of insulin are µIU/mL and units of glucose are mmol/mL.

To measure whole-body insulin sensitivity, the Matsuda index was calculated as follows, where higher values reflect increased insulin sensitivity [23]:

$$\:\mathrm{Matsuda\:Index}=\:\frac{\mathrm{10,000}}{\sqrt{\left(fasting\:glucose\:x\:fasting\:insulin\right)\:x\:\left(A*B\right)}}\:$$

where.

$$\:A=\:\frac{(fasting\:glucose*15+30\mathrm{min}glucose*30+60\mathrm{min}glucose*30+90\mathrm{min}glucose*30+120\mathrm{min}glucose*15)}{120}$$

and

$$\:B=\:\frac{(fasting\:insulin*15+30\mathrm{min}insulin*30+60\mathrm{min}insulin*30+90\mathrm{min}insulin*30+120\mathrm{min}insulin*15)}{120}$$

### MiRNAs

Circulating miRNAs were measured in baseline serum samples using NanoString [24]. The NanoString platform uses fluorescent barcodes bound to a capture probe to count 798 possible miRNAs. Details of the analytical process have been described previously [25]. After data processing and restricting the analysis to miRNA detected in more than 90% of participants, 142 miRNAs remained for analysis. miRNA values are expressed as normalized counts and log_2_ transformed.

### Proteomics

Proteins were measured at baseline in fasting plasma using the Olink Explore 384 Cardiometabolic panel, which uses a proximity extension assay to measure the relative abundance of 369 proteins [26]. Of these 369, 23 had 50% of observations below the limit of detection and were excluded from the analysis, leaving 346 proteins for analysis. Proteins are reported as normalized protein expression levels after log_2_ transformation.

### Metabolomics

Untargeted metabolomics were measured at baseline in plasma samples collected at the two-hour OGTT time point using liquid chromatography and high-resolution mass spectrometry (LCMS) methods as described previously [27]. Briefly, unique features were identified using mass-to-charge ratio (m/z), retention time, and peak intensity. Features were adjusted for batch variation and excluded if they were detected in < 75% of samples or if there was a > 30% coefficient of variability of the quality control samples after batch correction. After processing, the resulting 23,173 features were scaled to a standard normal distribution and log_2_ transformed. Of these 23,173 features, 466 confirmed compounds were identified using known standards. Metabolites’ identities were assigned by matching mass m/z (< 5ppm) and retention time (< 15 s). Where more than one molecule had retention times within the allowable error, the annotation with the closest retention time to the known standard was chosen.

### Covariates

Demographic characteristics were collected through questionnaires administered at baseline. Age was calculated from visit date and birthday. Participants self-reported their race and ethnicity (Non-Hispanic White, Hispanic, and Other), their sex, and if they exercised (yes or no). Participants also reported their parents’ highest level of education (less than high school, completed high school, more than high school, or don’t know). Body mass index (BMI, kg/m^2^) was calculated from clinical measurements of weight and height taken by trained personnel during the study visit and treated as a continuous measure in the analysis.

### Statistical analysis

Omics-wide association studies (OWAS) were performed to identify miRNAs, proteins, and metabolites associated with HEI at a nominal p-value of < 0.05 after adjusting for participant age, ethnicity, sex, and BMI. The proteomic and metabolomic OWAS results have been reported previously [28], while the miRNA OWAS results are reported in Table S1. Two miRNAs, miR-30a-5p and miR-377-3p, were associated with HEI and retained for the mediation analysis. Of the 346 measured proteins, 44 were associated with HEI and included in the mediation analysis. Nineteen annotated endogenous metabolites previously found to be nominally associated with HEI (*p* < 0.05) were also considered as possible mediators. All features considered for the mediation analysis are described in Table 1.

**Table 1:** Omics features associated with HEI and included in mediation analyses

miRNA	Proteins	Metabolites
miR-30a-5p, miR-377-3p	ACY1, ADAMTS16, ADH4, AGXT, ANG, CA5A, CANT1 CCL14, CCL15, CCL27, CDH2, CDHR5, CES1, COL18A1, CST3, CTSZ DPP7, F7, F9, GSTA1, HMOX1, HYOU1, ICAM1, IGSF8, IGALS1, LILRA5, LILRB1, LILRB2, NRP1, PDGFRA, PTPRS, RARRES2, SEMA3F, SERPINA11, SERPINB5, SIGLEC7, SOST, ST6GAL1, TFPI, THPO, TNFSF13B, VASN	Betaine; Heptanoate; Pipecolic Acid; Quinolinic Acid; Pyridoxine; Shikimate; L-Gulonolactone; 4-Pyridoxate; Undecylenic Acid; 5-HIAA; Indoxyl Sulfate; Biotin; 2-Methylbutyroylcarnitine; Hydroxybutyrylcarnitine; Stearidonic Acid; 5Z, 8Z, 11Z-Eicosatrienoic Acid; 7-Ketodeoxycholic Acid; Hyodeoxycholic Acid; Palmitoylcarnitine

Descriptive statistics (mean and standard deviation [SD] for continuous variables, frequency and percent for categorical variables) were calculated for exposure, outcomes, and covariates. Linear regression was used to evaluate the relationship between baseline HEI and all outcome variables measured at the follow up visit in separate models, each adjusting for age, sex, ethnicity, parental education, and exercise. Mediation analyses were then conducted for those exposure-outcome relationships with statistically significant (*p* < 0.05) associations. Missing values were considered to be missing completely at random, and no exposure, outcome, mediator, or covariate values were imputed. All analyses were performed using R Statistical Software (v4.3.1).

Multivariate mediation analyses were initially conducted using the “hima” package [29]. High dimensional mediation analysis (HIMA) is a three-step variable-selection approach to mediation with large numbers of potential variables (greater than or near sample size). This approach first excludes any variables not strongly associated with the outcome, then conducts further variable selection using a penalty (here, the minmax concave penalty). Finally joint significance testing is used to test for the mediation effect [29]. High dimensional mediation was conducted using an “early” integration approach, in which all omics layers are scaled, so that each feature has a mean of 0 and SD of 1, and combined into a single dataset, so that the strongest mediators across all layers are identified [30]. P-values were adjusted for a Benjamini-Hochberg false discovery rate (q-values), and selected features were considered to significantly mediate the relationship between exposure and outcome if q < 0.05. All omics features were normalized prior to inclusion in the mediation models.

Causal mediation analyses were then used to validate the individual features selected by HIMA and determine the direct effect of HEI on the outcome and the indirect effects of each mediator. Each feature was independently evaluated for mediation in separate models. Estimates and 95% confidence intervals (CIs) were calculated for the indirect (mediation) effect, direct effect, total effect, and proportion mediated using the “mediation” (v4.5.0) R package [31]. To determine whether the selected features from each mediation approach improved the fit of the linear regression model for HEI on the outcome, the selected omics features were added as additional covariates and the resulting R^2^s compared to the base model with no additional features. All mediation models adjusted for participants’ age at baseline, sex, ethnicity, parents’ education, and exercise. Mediation models assume that there is no unmeasured confounding between HEI and the outcome, mediators and the outcome, or HEI and no confounding between the mediators and outcome induced by HEI [32]. Product interaction terms were included to test for interaction between HEI and each mediator, as well as for interactions between mediators.

BMI is presumed to be on the causal pathway between diet and insulin sensitivity or type 2 diabetes, and thus was not included as a covariate in the primary analysis. A separate mediation analysis was performed for BMI to determine if it also might mediate the relationship between diet and our outcomes.

## Results

### Study population characteristics

Of the 85 participants who completed both study visits, 8 were missing metabolomics data, leaving 77 for analysis. At baseline, the average age of participants was 20 years old (SD = 1.2). The second visit took place approximately four years after the first (SD = 1.1 years). Participants had a mean baseline HEI score of 54.8 (SD = 13.2) out of a possible 100, were majority Hispanic, and about half were female (Table 2). Due to incomplete OGTT measurements, 3 participants were missing 2-hour glucose and 2-hour insulin, and 5 participants were missing Matsuda Index scores at follow up (Figure S1).

**Table 2:** Descriptive statistics for participant demographics, exposure, and outcomes

Variable	*n* = 77
Sex, n (%)	
Female	40 (51.9%)
Male	37 (48.1%)
Ethnicity, n (%)	
Hispanic/Latino	44 (57.1%)
Non-Hispanic White	28 (36.4%)
Other	5 (6.5%)
Parental Education, n (%)	
Did not complete high school	13 (16.9%)
Completed high school	10 (13.0%)
More than high school	52 (67.5%)
Don’t know	2 (2.6%)
Exercise, n (%)	
Yes	59 (76.6%)
No	18 (23.4%)
Baseline Age (years), Mean (SD)	20.0 (1.2)
Follow Up Time (years), Mean (SD)	4.0 (1.1)
BMI (kg/m^2^), Mean (SD)	29.9 (4.9)
HEI, Mean (SD)	54.8 (13.2)
Fasting Glucose (mg/dL), Mean (SD)	95.4 (17.2)
2-Hour Glucose (mg/dL), Mean (SD)	120 (35.8)
Missing, %	3 (3.9%)
Fasting Insulin (mcU/L), Mean (SD)	13.0 (11.5)
2-Hour Insulin (mcU/L), Mean (SD)	73.5 (70.5)
Missing, n (%)	3 (3.9%)
HOMA-β, Mean (SD)	146 (123)
HOMA-IR, Mean (SD)	3.28 (3.86)
Matsuda Index, Mean (SD)	4.53 (2.85)
Missing, n (%)	5 (6.5%)

BMI, HEI, and exercise were assessed at baseline. Glucose, insulin, HOMA, and Matsuda index measures were obtained at follow-up.

Standard deviation, *SD* Body mass index, *BMI* Healthy Eating Index, *HEI* homeostatic model assessment, *HOMA* insulin resistance, IR

### Linear regression

HEI was inversely, but not significantly, associated with 2-hour glucose, fasting insulin, 2-hour insulin, HOMA-IR, and HOMA-β (Table 3). We also observed positive but non-significant associations between HEI and fasting glucose. However, HEI was significantly positively associated with Matsuda Index: one point increase in HEI was associated with a 0.051 (95% CI: 0.0035, 0.098) unit increase in Matsuda Index, indicating that higher HEI scores at baseline were linked to higher insulin sensitivity at the follow up visit. As this was the only relationship to achieve statistical significance, the association between HEI and Matsuda Index was the only one further investigated for mediation by omics features.

**Table 3:** Results from linear regressions between baseline HEI and insulin- and glucose-related outcomes at follow up

Outcome	*n*	Beta	SE	*P*-Value
Fasting glucose (mg/dL)	77	0.101	0.152	0.510
2-Hour glucose (mg/dL)	74	−0.215	0.322	0.507
Fasting insulin (mcU/L)	77	−0.090	0.094	0.341
2-Hour Insulin (mcU/L)	74	−0.606	0.586	0.305
HOMA-β	77	−1.089	0.999	0.279
HOMA-IR	77	−0.004	0.033	0.894
Matsuda Index	72	0.051	0.024	0.036

All models adjusted for participants’ age at baseline, sex, ethnicity, parental education, and exercise

Healthy Eating Index, *HEI* homeostatic model assessment, *HOMA* insulin resistance, *IR* standard error, SE

### High-dimensional mediation and feature selection

**Fig. 1:**
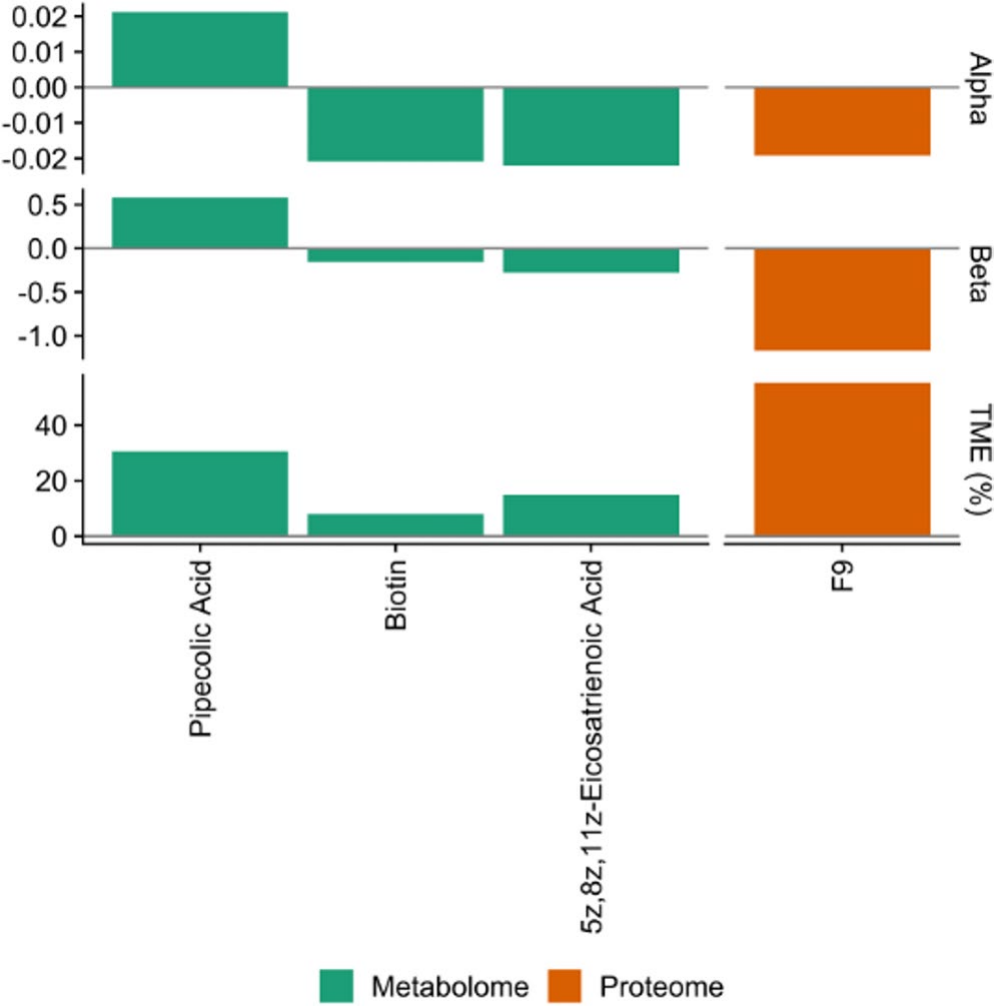
Significant (q < 0.05) features selected using high-dimensional mediation analysis. Alpha is the estimate for the association of the HEI on each feature (M ~ X + covariates), beta is the estimate for the association of each feature on Matsuda Index adjusted for HEI (Y ~ M + X + covariate), and TME (%) is the percent of the total effect mediated by each selected feature

HIMA selected four metabolites and two proteins: pipecolic acid, indoxyl sulfate, biotin, 5Z, 8Z, 11Z-eicosatrienoic acid, F9, and PDGFRA. Four of these features (pipecolic acid, biotin, 5Z, 8Z, 11Z-eicosatrienoic acid, and F9) were significant mediators (q < 0.05) (Table S2). F9 (β_HEI_ = −0.019, β_Matsuda_ = −1.17), biotin (β_HEI_ = −0.021, β_Matsuda_ = −0.15), and 5Z, 8Z, 11Z-eicosatrienoic acid (β_HEI_ = −0.022, β_Matsuda_ = −0.28) were inversely associated with both HEI and Matsuda Index, while pipecolic acid (β_HEI_ = 0.021, β_Matsuda_ = 0.58) was positively associated with both HEI and Matsuda Index. All significant features were associated with an overall positive indirect effect. F9 (coagulation factor IX) and pipecolic acid contributed the most to the total effect of HEI on Matsuda Index (55.3% and 30.5%, respectively) (Fig. 1). No miRNAs were selected as mediators.

### Causal mediation

**Fig. 2:**
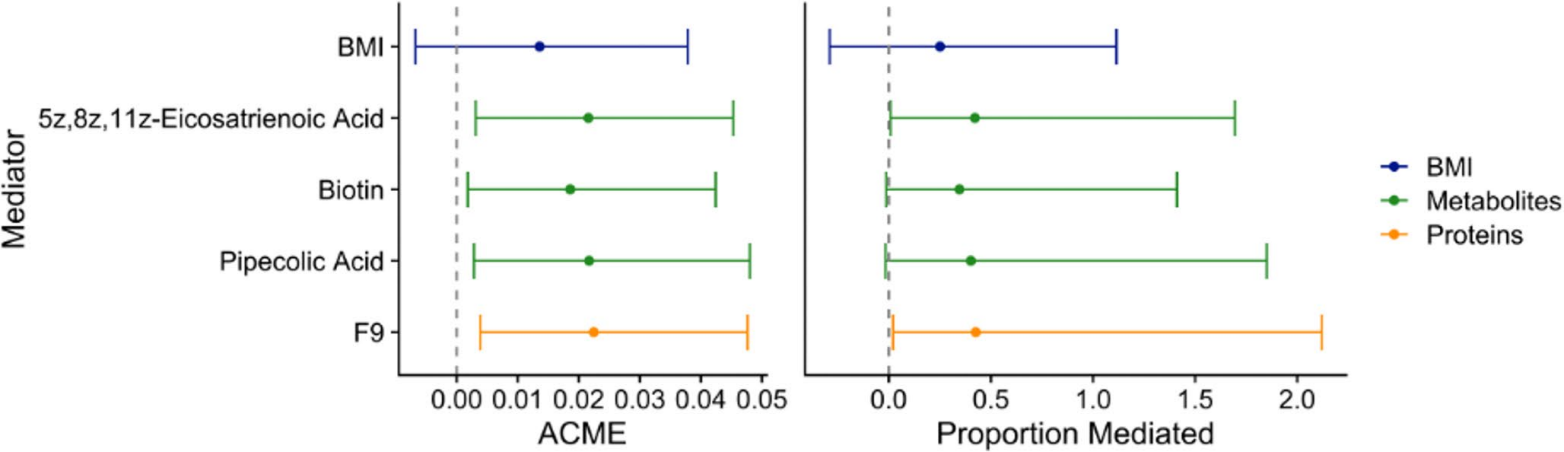
Effect estimates and 95% confidence intervals from causal mediation analysis for HIMA-selected omics features and BMI. ACME is the average causal mediation effect, for each one unit increase in HEI. Analyses adjusted for age, sex, ethnicity, parental education, and exercise

Causal mediation analyses were conducted independently with each of the four significant features selected by HIMA. The indirect effects of each feature were positive, and each feature independently mediated between 35 and 43% for the total effect of HEI on Matsuda Index (Fig. 2, Table S3). BMI was not found to be a statistically significant independent mediator. No statistically significant interactions were detected between HEI and F9 (β_interaction_ = −0.027, p-value = 0.12), pipecolic acid (β_interaction_ = 0.019, p-value = 0.46), biotin (β_interaction_ = −0.0047, p-value = 0.85), or 5Z, 8Z, 11Z-eicosatrienoic acid (β_interaction_ = −0.016, p-value = 0.48).

In the base linear regression model for the relationship between HEI and Matsuda Index, adjusting for covariates, the adjusted R^2^ was 0.18. After adding F9, pipecolic acid, biotin, and 5Z, 8Z, 11Z-eicosatrienoic acid to the model, the adjusted R^2^ increased to 0.50 (Table 4) and did not change after further adjusting for BMI. A likelihood ratio test indicated that the addition of the selected metabolites and protein significantly improved the model fit (*p* < 0.0001). The direct effect of HEI on Matsuda Index, after adjusting for covariates and the four mediating omics features, is −0.012 (95% CI: −0.054, 0.029), suggesting that the effect of HEI on insulin sensitivity is completely mediated by F9, biotin, pipecolic acid, and 5Z, 8Z, 11Z-eicosatrienoic acid. No statistically significant interactions between any of the mediators were observed (Table S4).

**Table 4:** Comparisons between linear regression models with and without mediators

	Beta (HEI)	95% CI		DF	*R* ^2^	Chi^2^	LRT *p*-value
		Lower	Upper				
Model 1	0.051	0.0035	0.098	65	0.18	-	-
Model 2	−0.012	−0.054	0.029	61	0.5	39.64	< 0.0001
Model 3	0.038	−0.0062	0.081	64	0.32	14.41	0.00015
Model 4	−0.01	−0.052	0.032	60	0.5	41.17	< 0.0001
Model 1: Matsuda Index ~ HEI + age + sex + parental education + exercise + ethnicity							
Model 2: Model 1 + F9, 5z,8z,11z-eicosatrienoic acid, biotin, pipecolic acid							
Model 3: Model 1 + BMI							
Model 4: Model 1 + BMI + F9, 5z,8z,11z-eicosatrienoic acid, biotin, pipecolic acid							

*HEI* Healthy Eating Index 2015, *CI* Confidence Interval, *DF* Degrees of freedom, *LRT* Likelihood ratio test for each model compared to Model 1

## Discussion

In this study, we examined for the first time the potential for miRNAs, proteomics features, and metabolomics features to mediate the relationship between diet quality and insulin sensitivity. We identified four omics features, 5z,8z,11z-eicosatrienoic acid (or mead acid, a polyunsaturated fatty acid), biotin (a B-vitamin), pipecolic acid (an amino acid), and F9 (a protein involved in blood clotting), which each independently mediated between 30 and 43% of the total effect between HEI scores and insulin sensitivity in a cohort of young adults. Accounting for these biomarkers in a linear model with HEI and other risk factors (demographics, exercise) substantially improved the model fit and explained much more of the variability in the Matsuda index than HEI alone. BMI was not found to significantly modify this relationship. This novel analysis is the first, to our knowledge, to examine multiple omics layers for mediation between diet and insulin sensitivity.

Despite interest in the application of omics technologies to nutritional epidemiology [11, 33] and precision medicine [34], few studies have considered the mediating effects of omics features in established relationships between diet and disease. The mechanisms underlying these relationships are likely numerous and not yet completely understood, though our results provide evidence that metabolic and proteomic alterations resulting from dietary intake may be responsible for the biological changes that lead to insulin insensitivity, glucose intolerance, and eventually type 2 diabetes. The four mediators identified in this analysis, F9, biotin, pipecolic acid, and mead acid, have previously been linked to type 2 diabetes or insulin resistance and may play an important role in the development of metabolic disease [35–38].

Two features, F9 and mead acid were inversely associated with HEI and insulin sensitivity and appear to be elevated in plasma in response to inflammation. F9 is a critical protein involved in hemostasis and has been identified in previous proteomics analyses as a possible biomarker for type 2 diabetes and related conditions [38–40]. Coagulation cascade pathways, which include F9, are associated with beta cell function [40] and are activated in response to increased inflammation [41]. Mead acid is an endogenous n-9 polyunsaturated fatty acid, is detected in high levels in the presence of essential fatty acid (EFA) deficiency [42], and previously has been found to be associated with increased risk for type 2 diabetes [37]. Poor diet quality may contribute to insufficient intake of EFAs or disrupt the balance of omega-3 and omega-6 fatty acids [43], which may result in increased production of mead acid. This may increase inflammation, which then leads to insulin resistance in muscle and hepatic cells [44]. Biotin (vitamin B7), was also inversely associated with both HEI and insulin sensitivity. To our knowledge, no previous associations between circulating biotin and any dietary patterns have been reported. However, biotin is positively associated with anti-inflammatory functions and both in vitro studies and clinical trials have suggested that biotin supplementation may play a role in the prevention and management of type 2 diabetes [35]. Pipecolic acid, positively associated with both HEI and insulin sensitivity in this study, is linked to increased consumption of fruits and vegetables [45, 46]. In contrast to our finding with insulin sensitivity, pipecolic acid has been associated with increased risk for type 2 diabetes or type 2 diabetes comorbidities in previous work [36, 47], though few studies have investigated this relationship. This apparent contradiction requires further exploration in future studies.

Though the emergence of omics technologies offers the opportunity to identify new potential biomarkers, mechanisms, or drug targets, these analyses are still largely exploratory. Existing research supports links between each of the four features identified in this analysis with dietary exposures and insulin sensitivity or diabetes, but there is limited information available about the biological mechanisms connecting these molecules. For instance, while it does not appear that any research to date has reported a direct biological link between F9 and mead acid, in vitro studies have shown that mead acid can affect platelet aggregation [48], though it is not clear if this occurs through pathways involving F9. We found that pipecolic acid may have a protective effect on insulin sensitivity and type 2 diabetes, but the only previous study, to our knowledge, to also evaluate pipecolic acid with respect to type 2 diabetes risk factors found the opposite [36]. Additionally, though the three metabolites identified in this analysis do not belong to the same metabolic pathways, all metabolites and F9 appear to be involved in pro- or anti- inflammatory responses.

The most significant strength of this study is the integration of multiple omics layers, which has allowed us to identify a collection of molecules that mediate the established relationship between diet quality and insulin sensitivity. Other strengths include the longitudinal study design, meaning that these findings are not the result of reverse causation, and the use of the Matsuda index calculated from five OGTT time points, which is an indicator of combined hepatic and peripheral tissue sensitivity to insulin [23]. The Matsuda index captures more information on overall insulin resistance, regardless of an individual’s unique pathology or disease progression, and is comparable to gold standard measurements for insulin sensitivity such as the euglycemic-hyperinsulinemic clamp [49]. Additionally, this study was conducted in a population frequently underrepresented in biomedical research. As insulin sensitivity may differ across racial and ethnic groups [50], this study provides evidence for mediation by omics biomarkers in a primarily Hispanic cohort. Agreement between our mediation results and previous work identifying biomarkers of type 2 diabetes suggests that these results may be generalizable across ethnicities and age of type 2 diabetes onset, though additional validation is needed.

This study also has several limitations. Our sample size is small, which limited our ability to detect significant associations for both our exposure-outcome relationships and mediation analyses. There may additional mediators between diet and insulin sensitivity that we did not have the power to detect. However, we applied a strict FDR correction for mediator selection to limit spurious findings, and our selected mediators are biologically plausible. Additionally, our assumption that the diet and omics measures reported here are indicative of long-term diet and long-term biological processes may not be correct, though this is a common assumption in both nutritional and molecular epidemiology. We also recognize the limitations of mediation analysis, especially in its relatively new application to high-dimensional data. In this analysis, we have assumed that there is no unmeasured confounding between our exposure and mediators, the mediators and outcome, or between our exposure and outcome. Because many factors may influence omics measurements, including environmental factors, pharmaceuticals, or genetics, this too may not be valid. Finally, an additional consideration for high dimensional mediation is the assumption that there is no influence by one mediator on another; while we have no specific biological evidence for a relationship between these four features and we did not observe any statistical interaction, biological systems are complex and we cannot fully exclude the possibility.

## Conclusions

Four omics features, F9, pipecolic acid, biotin, and 5z,8z,11z-eicosatrienoic acid, were found to be significant mediators of the relationship between diet quality and insulin sensitivity. These mediators have been previously associated with type 2 diabetes in adults and may be involved in the inflammatory responses that are though to underly the development of insulin resistance. While the sample size in the study is small, and these findings should be considered preliminary, these results identify possible biomarkers for insulin sensitivity that could be assessed over the course of disease development to monitor diet adherence or other preventive interventions for young-onset type 2 diabetes.

## Supplementary Information

Below is the link to the electronic supplementary material.

Supplementary Material 1 (DOCX 62.9 KB)

## Data Availability

The datasets generated during and/or analyzed during the current study are not publicly available due to individual privacy concerns but may be available from the corresponding author on reasonable request.
