# Supplementary material for: Metabolites and proteins may mediate the relationship between diet quality and insulin sensitivity in young adult cohort
Source: J Diabetes Metab Disord. 2026 Feb 26;25(1):90. doi: 10.1007/s40200-026-01918-3 (PMC12946319; doi:10.1007/s40200-026-01918-3)
Supplement: Supplementary file 1 — Supplementary Material 1 (DOCX 62.9 KB) [file 40200_2026_1918_MOESM1_ESM.docx]

Supplementary Material


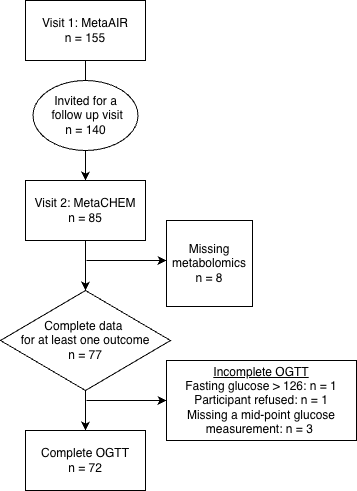


Figure S1. Flowchart for participant enrollment and data collection. Abbreviations: oral glucose tolerance test, OGTT.

Table S1. Results from an omics-wide association study between miRNA and the Healthy Eating Index 2015.

| miRNA | Beta | SE | P-value |
| --- | --- | --- | --- |
| hsa-let-7a-5p | -0.007 | 0.004 | 0.106 |
| hsa-let-7b-5p | 0.007 | 0.005 | 0.157 |
| hsa-let-7c-5p | -0.006 | 0.006 | 0.282 |
| hsa-let-7d-5p | -0.003 | 0.006 | 0.543 |
| hsa-let-7e-5p | -0.018 | 0.009 | 0.053 |
| hsa-let-7f-5p | -0.004 | 0.008 | 0.621 |
| hsa-let-7g-5p | 0.000 | 0.003 | 0.976 |
| hsa-let-7i-5p | -0.004 | 0.019 | 0.831 |
| hsa-miR-106a-5p-hsa-miR-17-5p | 0.003 | 0.004 | 0.472 |
| hsa-miR-106b-5p | 0.005 | 0.006 | 0.418 |
| hsa-miR-107 | -0.010 | 0.010 | 0.318 |
| hsa-miR-1183 | -0.001 | 0.011 | 0.941 |
| hsa-miR-122-5p | -0.011 | 0.012 | 0.382 |
| hsa-miR-1246 | -0.018 | 0.013 | 0.157 |
| hsa-miR-1255a | 0.006 | 0.014 | 0.649 |
| hsa-miR-125a-5p | -0.012 | 0.008 | 0.125 |
| hsa-miR-125b-5p | 0.004 | 0.009 | 0.689 |
| hsa-miR-126-3p | -0.006 | 0.004 | 0.101 |
| hsa-miR-1260b | 0.012 | 0.008 | 0.131 |
| hsa-miR-127-3p | 0.007 | 0.009 | 0.462 |
| hsa-miR-128-3p | 0.009 | 0.008 | 0.294 |
| hsa-miR-1285-3p | -0.003 | 0.012 | 0.827 |
| hsa-miR-1299 | 0.002 | 0.009 | 0.841 |
| hsa-miR-130a-3p | -0.004 | 0.005 | 0.448 |
| hsa-miR-132-3p | 0.008 | 0.010 | 0.437 |
| hsa-miR-136-5p | -0.003 | 0.010 | 0.789 |
| hsa-miR-140-5p | 0.009 | 0.007 | 0.246 |
| hsa-miR-142-3p | -0.006 | 0.006 | 0.269 |
| hsa-miR-144-3p | 0.013 | 0.009 | 0.150 |
| hsa-miR-145-5p | 0.004 | 0.009 | 0.681 |
| hsa-miR-146a-5p | -0.004 | 0.007 | 0.547 |
| hsa-miR-148a-3p | -0.010 | 0.005 | 0.057 |
| hsa-miR-148b-3p | -0.012 | 0.007 | 0.096 |
| hsa-miR-150-5p | 0.007 | 0.006 | 0.280 |
| hsa-miR-151a-3p | 0.006 | 0.007 | 0.378 |
| hsa-miR-151a-5p | 0.005 | 0.008 | 0.493 |
| hsa-miR-1537-3p | 0.001 | 0.008 | 0.944 |
| hsa-miR-15a-5p | -0.005 | 0.005 | 0.331 |
| hsa-miR-15b-5p | -0.002 | 0.004 | 0.567 |
| hsa-miR-16-5p | 0.007 | 0.006 | 0.260 |
| hsa-miR-181a-5p | -0.007 | 0.006 | 0.242 |
| hsa-miR-185-5p | -0.003 | 0.006 | 0.659 |
| hsa-miR-186-5p | 0.007 | 0.008 | 0.426 |
| hsa-miR-18a-5p | -0.002 | 0.007 | 0.751 |
| hsa-miR-190a-5p | 0.008 | 0.010 | 0.402 |
| hsa-miR-191-5p | -0.001 | 0.004 | 0.898 |
| hsa-miR-1910-5p | 0.009 | 0.008 | 0.271 |
| hsa-miR-193b-3p | 0.016 | 0.011 | 0.147 |
| hsa-miR-196a-5p | 0.007 | 0.011 | 0.533 |
| hsa-miR-197-3p | -0.014 | 0.008 | 0.076 |
| hsa-miR-199a-3p-hsa-miR-199b-3p | -0.009 | 0.005 | 0.104 |
| hsa-miR-199a-5p | -0.012 | 0.006 | 0.060 |
| hsa-miR-199b-5p | 0.002 | 0.011 | 0.843 |
| hsa-miR-19a-3p | 0.003 | 0.007 | 0.698 |
| hsa-miR-19b-3p | 0.008 | 0.005 | 0.108 |
| hsa-miR-20a-5p-hsa-miR-20b-5p | 0.006 | 0.005 | 0.195 |
| hsa-miR-21-5p | -0.002 | 0.006 | 0.741 |
| hsa-miR-22-3p | 0.002 | 0.004 | 0.577 |
| hsa-miR-221-3p | -0.011 | 0.007 | 0.114 |
| hsa-miR-222-3p | 0.001 | 0.005 | 0.814 |
| hsa-miR-223-3p | -0.002 | 0.004 | 0.538 |
| hsa-miR-23a-3p | 0.002 | 0.004 | 0.669 |
| hsa-miR-23b-3p | 0.004 | 0.008 | 0.656 |
| hsa-miR-24-3p | 0.003 | 0.007 | 0.669 |
| hsa-miR-25-3p | 0.008 | 0.006 | 0.162 |
| hsa-miR-26a-5p | -0.010 | 0.008 | 0.189 |
| hsa-miR-26b-5p | 0.006 | 0.006 | 0.318 |
| hsa-miR-27a-3p | -0.006 | 0.010 | 0.520 |
| hsa-miR-27b-3p | -0.010 | 0.006 | 0.117 |
| hsa-miR-28-5p | -0.011 | 0.008 | 0.174 |
| hsa-miR-299-3p | 0.005 | 0.011 | 0.679 |
| hsa-miR-299-5p | 0.006 | 0.011 | 0.571 |
| hsa-miR-29a-3p | 0.005 | 0.009 | 0.605 |
| hsa-miR-29b-3p | -0.010 | 0.006 | 0.108 |
| hsa-miR-29c-3p | -0.001 | 0.006 | 0.894 |
| hsa-miR-301a-3p | -0.011 | 0.009 | 0.217 |
| hsa-miR-302d-3p | 0.007 | 0.017 | 0.687 |
| hsa-miR-30a-5p | 0.019 | 0.008 | 0.020 |
| hsa-miR-30b-5p | -0.004 | 0.009 | 0.692 |
| hsa-miR-30c-5p | 0.003 | 0.008 | 0.697 |
| hsa-miR-30d-5p | 0.000 | 0.007 | 0.975 |
| hsa-miR-30e-3p | 0.003 | 0.009 | 0.748 |
| hsa-miR-30e-5p | 0.003 | 0.008 | 0.751 |
| hsa-miR-32-5p | 0.002 | 0.007 | 0.811 |
| hsa-miR-320e | -0.001 | 0.010 | 0.899 |
| hsa-miR-323a-3p | 0.000 | 0.009 | 0.983 |
| hsa-miR-323b-3p | 0.003 | 0.011 | 0.745 |
| hsa-miR-335-5p | 0.010 | 0.009 | 0.270 |
| hsa-miR-337-3p | -0.006 | 0.010 | 0.550 |
| hsa-miR-337-5p | 0.000 | 0.010 | 0.962 |
| hsa-miR-340-5p | -0.001 | 0.008 | 0.902 |
| hsa-miR-342-3p | 0.000 | 0.006 | 0.934 |
| hsa-miR-361-3p | -0.003 | 0.008 | 0.746 |
| hsa-miR-361-5p | -0.001 | 0.008 | 0.887 |
| hsa-miR-363-3p | 0.007 | 0.009 | 0.469 |
| hsa-miR-367-3p | 0.008 | 0.011 | 0.484 |
| hsa-miR-374a-5p | -0.006 | 0.004 | 0.157 |
| hsa-miR-374b-5p | -0.014 | 0.007 | 0.060 |
| hsa-miR-376a-3p | 0.006 | 0.008 | 0.433 |
| hsa-miR-376c-3p | 0.002 | 0.009 | 0.813 |
| hsa-miR-377-3p | 0.017 | 0.009 | 0.045 |
| hsa-miR-378g | -0.010 | 0.011 | 0.343 |
| hsa-miR-378i | -0.006 | 0.011 | 0.566 |
| hsa-miR-379-5p | 0.009 | 0.010 | 0.391 |
| hsa-miR-382-5p | 0.000 | 0.009 | 0.999 |
| hsa-miR-409-3p | 0.014 | 0.008 | 0.076 |
| hsa-miR-421 | 0.021 | 0.011 | 0.053 |
| hsa-miR-423-3p | -0.006 | 0.009 | 0.477 |
| hsa-miR-423-5p | 0.000 | 0.004 | 0.954 |
| hsa-miR-424-5p | 0.001 | 0.009 | 0.893 |
| hsa-miR-425-5p | 0.010 | 0.007 | 0.140 |
| hsa-miR-432-5p | -0.005 | 0.010 | 0.618 |
| hsa-miR-4421 | 0.009 | 0.011 | 0.379 |
| hsa-miR-4454-hsa-miR-7975 | -0.016 | 0.009 | 0.081 |
| hsa-miR-450a-5p | 0.003 | 0.009 | 0.767 |
| hsa-miR-451a | 0.009 | 0.008 | 0.264 |
| hsa-miR-454-3p | 0.005 | 0.009 | 0.538 |
| hsa-miR-485-3p | -0.006 | 0.009 | 0.518 |
| hsa-miR-486-3p | -0.007 | 0.009 | 0.426 |
| hsa-miR-487b-3p | 0.011 | 0.009 | 0.217 |
| hsa-miR-491-5p | 0.000 | 0.010 | 0.986 |
| hsa-miR-495-3p | -0.006 | 0.011 | 0.577 |
| hsa-miR-503-5p | 0.008 | 0.009 | 0.383 |
| hsa-miR-506-3p | 0.000 | 0.010 | 0.998 |
| hsa-miR-513b-5p | 0.006 | 0.009 | 0.486 |
| hsa-miR-514a-5p | 0.013 | 0.011 | 0.232 |
| hsa-miR-518b | 0.004 | 0.008 | 0.661 |
| hsa-miR-526a-hsa-miR-518c-5p-hsa-miR-518d-5p | -0.006 | 0.011 | 0.595 |
| hsa-miR-543 | -0.009 | 0.010 | 0.359 |
| hsa-miR-548d-5p | 0.005 | 0.010 | 0.623 |
| hsa-miR-590-5p | 0.016 | 0.009 | 0.075 |
| hsa-miR-597-5p | -0.003 | 0.012 | 0.839 |
| hsa-miR-598-3p | 0.010 | 0.010 | 0.363 |
| hsa-miR-612 | 0.009 | 0.011 | 0.400 |
| hsa-miR-652-3p | 0.007 | 0.007 | 0.306 |
| hsa-miR-660-5p | 0.006 | 0.009 | 0.537 |
| hsa-miR-664a-3p | 0.005 | 0.009 | 0.577 |
| hsa-miR-7-5p | 0.012 | 0.011 | 0.281 |
| hsa-miR-92a-3p | 0.010 | 0.007 | 0.143 |
| hsa-miR-93-5p | 0.006 | 0.004 | 0.097 |
| hsa-miR-98-5p | -0.012 | 0.008 | 0.151 |
| hsa-miR-99b-5p | 0.003 | 0.009 | 0.722 |
| Notes: All analyses adjusted for age, sex, ethnicity, and BMI | | | |

Table S2. HIMA results for selected omics features.

| Omic Layer | Feature | Alpha | Beta | Indirect Effect | TME (%) | q-value |
| --- | --- | --- | --- | --- | --- | --- |
| Metabolome | Pipecolic Acid | 0.021 | 0.58 | 0.012 | 30.54 | 0.036 |
|  | Indoxyl Sulfate | -0.0069 | -0.27 | 0.0019 | 4.56 | 0.43 |
|  | Biotin | -0.021 | -0.15 | 0.0032 | 7.93 | 0.038 |
|  | 5z,8z,11z-Eicosatrienoic Acid | -0.022 | -0.28 | 0.0061 | 14.96 | 0.036 |
| Proteome | F9 | -0.019 | -1.17 | 0.022 | 55.30 | 0.036 |
|  | PDGFRA | -0.0082 | 0.66 | -0.0054 | -13.30 | 0.43 |

Notes: Alpha is the estimate for the association of the HEI on each feature adjusting for covariates, beta is the estimate for the association of each feature on Matsuda Index adjusted for the exposure and covariates, TME (%) is the percent of the total effect mediated by each selected feature.

Table S3. Results from causal mediation analyses for HIMA-selected features and BMI.

|  | ACME  β (95% CI) | ADE  β (95% CI) | Proportion Mediated  β (95% CI) |
| --- | --- | --- | --- |
| F9 | 0.023 (0.0039, 0.049) | 0.027 (-0.020, 0.073) | 0.43 (-0.0030, 2.38) |
| 5z,8z,11z-Eicosatrienoic Acid | 0.022 (0.0033, 0.046) | 0.028 (-0.017, 0.072) | 0.41 (-0.058, 1.95) |
| Biotin | 0.019 (0.0028, 0.043) | 0.032 (-0.014, 0.076) | 0.35 (-0.18, 1.57) |
| Pipecolic Acid | 0.21 (0.0025, 0.049) | 0.029 (-0.018, 0.074) | 0.41 (-0.085, 2.16) |
| BMI | 0.013 (-0.0055, 0.038) | 0.038 (-0.0036, 0.082) | 0.25 (-0.21, 1.03) |

Abbreviations: Confidence interval, CI; average causal mediation effect, ACME; average direct effect, ADE; body mass index, BMI

Table S4. Product interaction terms between each of the selected mediators.

| Interaction Term | β_interaction_ | SE | p-value |
| --- | --- | --- | --- |
| Pipecolic Acid*5z,8z,11z-Eicosatrienoic Acid | -0.070 | 0.27 | 0.80 |
| Pipecolic Acid*Biotin | -0.55 | 0.36 | 0.14 |
| 5z,8z,11z-Eicosatrienoic Acid*Biotin | 0.20 | 0.32 | 0.55 |
| Pipecolic Acid*F9 | -0.19 | 0.30 | 0.54 |
| 5z,8z,11z-Eicosatrienoic Acid*F9 | -0.77 | 0.38 | 0.051 |
| Biotin*F9 | 0.25 | 0.32 | 0.43 |
| Pipecolic Acid*5z,8z,11z-Eicosatrienoic Acid*Biotin | 0.038 | 0.32 | 0.91 |
| Pipecolic Acid*5z,8z,11z-Eicosatrienoic Acid*F9 | -0.52 | 0.38 | 0.18 |
| Pipecolic Acid*Biotin*F9 | 0.63 | 0.37 | 0.10 |
| 5z,8z,11z-Eicosatrienoic Acid*Biotin*F9 | -0.18 | 0.34 | 0.60 |
| Pipecolic Acid*5z,8z,11z-Eicosatrienoic Acid*Biotin*F9 | -0.36 | 0.35 | 0.30 |

Model: Matsuda Index ~ HEI + covariates + F9*5z,8z,11z-eicosatrienoic acid*biotin*pipecolic acid

Abbreviations: Standard error, SE.
